# The biofilm-associated surface protein Esp of *Enterococcus faecalis* forms amyloid-like fibers

**DOI:** 10.1038/s41522-020-0125-2

**Published:** 2020-03-27

**Authors:** Agustina Taglialegna, Leticia Matilla-Cuenca, Pedro Dorado-Morales, Susanna Navarro, Salvador Ventura, James A. Garnett, Iñigo Lasa, Jaione Valle

**Affiliations:** 1 Instituto de Agrobiotecnología (IDAB), CSIC-UPNA-Gobierno de Navarra, Avenida Pamplona 123, Mutilva-31192, Navarra, Spain; 2 Navarrabiomed-Universidad Pública de Navarra-Departamento de Salud, IDISNA, Pamplona-31008, Navarra, Spain; 3 Institut de Biotecnologia i de Biomedicina and Departament de Bioquimica i Biologia Molecular, Universitat Autonoma de Barcelona, Bellaterra, Spain; 4 Centre for Host Microbiome Interactions, Dental institute, King’s College London, London, UK

**Keywords:** Biofilms, Microbiome

## Abstract

Functional amyloids are considered as common building block structures of the biofilm matrix in different bacteria. In previous work, we have shown that the staphylococcal surface protein Bap, a member of the Biofilm-Associated Proteins (BAP) family, is processed and the fragments containing the N-terminal region become aggregation-prone and self-assemble into amyloid-like structures. Here, we report that Esp, a Bap-orthologous protein produced by *Enterococcus faecalis*, displays a similar amyloidogenic behavior. We demonstrate that at acidic pH the N-terminal region of Esp forms aggregates with an amyloid-like conformation, as evidenced by biophysical analysis and the binding of protein aggregates to amyloid-indicative dyes. Expression of a chimeric protein, with its Esp N-terminal domain anchored to the cell wall through the R domain of clumping factor A, showed that the Esp N-terminal region is sufficient to confer multicellular behavior through the formation of an extracellular amyloid-like material. These results suggest that the mechanism of amyloid-like aggregation to build the biofilm matrix might be widespread among BAP-like proteins. This amyloid-based mechanism may not only have strong relevance for bacteria lifestyle but could also contribute to the amyloid burden to which the human physiology is potentially exposed.

## Introduction

In the last two decades, an increasing amount of studies have shown that amyloid structures are not restricted to protein aggregation disorders but also, they can be found as functional amyloids for a number of relevant biological processes in eukaryotic cells, fungi, and bacteria^1,2^. In bacteria, one of the most predominant processes in which functional amyloids have a biological role is in the context of multicellular behavior and biofilm formation. Biofilms are the most common bacterial growth in natural environments, medical, and industrial settings^3^. In biofilms, bacteria are embedded in an extracellular matrix that provides structural integrity, protects bacteria from the action of the immune system and antimicrobial treatments, favors nutrient adsorption and keeps extracellular enzymes close to the cells^4^. Amyloids are especially well suited for assembling the biofilm matrix in the extracellular environment because they provide a stable chassis for its inherent resistance against protease digestion and denaturation^5^. Amyloids can also modify the viscoelastic properties of the extracellular matrices helping biofilms to adapt and respond to environmental fluctuations^6^. Therefore, proteins with amyloid or amyloid-like conformation have been proven widespread in biofilms of many unrelated bacteria^7^. The most studied and characterized bacterial amyloid system is the curli fimbriae present in *Escherichia coli* and in other enterobacterial species, which is composed of the major structural subunit CsgA^8,9^. Other examples of biofilm-related amyloids are FapC in *Pseudomonas*^10^, TasA in *Bacillus subtilis*^11^, chaplins in *Streptomyces coelicolor*^12^ or the phenol-soluble modulins (PSMs) in the pathogenic bacteria *Staphylococcus aureus*^13^. In all these examples, functional amyloid assembly involves specialized machinery that controls the expression, secretion and polymerization of protein subunits. A more simplistic type of amyloid-like material is the one formed by the biofilm-associated protein (Bap) of *S. aureus*^14^. This surface protein undergoes partial proteolytic cleavage that releases fragments containing the N-terminal region of Bap only under specific environmental conditions^14^. This region has a molten globule-like conformation that, when the pH becomes acidic, switches to a β-sheet-rich conformation and polymerizes to form amyloid-like fibers. The N-terminal region of Bap contains two EF-hand calcium-binding motifs. Binding of calcium to the EF-hand motifs stabilizes the molten globule conformation. This prevents β-sheet transition and the subsequent self-assembly into amyloid fibers, hindering biofilm formation. Therefore, inactivation of the EF-hand calcium-binding domains by site-directed mutagenesis did not affect biofilm formation in the presence of calcium^14,15^.

The enterococcal surface protein Esp is a Bap-orthologous protein^16,17^. Esp is a high molecular weight protein of 1873 amino acids. It contains well-differentiated modules including the N-terminal domain, sharing 26% sequence identity with Bap; the central core region with two domains (A and B) and a series of tandem repeats sharing 23% sequence identity with C-repeats of Bap; and the C-terminal domain that includes the LPXTG-like motif found in surface-associated proteins. Esp contributes to biofilm formation by *E. faecalis*. It was demonstrated that the minimal region contributing to Esp-mediated biofilm enhancement was confined to the N-terminal domain^17^. However, the molecular mechanisms by which the N-terminal region of Esp performs this function is unknown. Based in our previous results obtained with Bap of *S. aureus*, we hypothesize that these proteins could mediate intercellular connection through a common molecular mechanism of amyloid-like conformation. By using biophysical in vitro assays, cell-based and microscopic approaches and dye-binding analyses, we demonstrated that the N-terminal domain of Esp formed aggregates with amyloid-like properties when the pH of the media became acidic. We further showed that the N-terminal domain of Esp is functional as it was able to induce multicellular behavior in bacteria lacking the *esp* gene through the formation of amyloid-like material, both when it was exogenously added to the culture and when it was expressed from a plasmid. Overall, our results suggest that the mechanism of amyloid-like aggregation might be a widespread mechanism of the BAP-like proteins to build the biofilm matrix.

## Results

### Computational algorithms predict Esp aggregation tendency

**Fig. 1:**
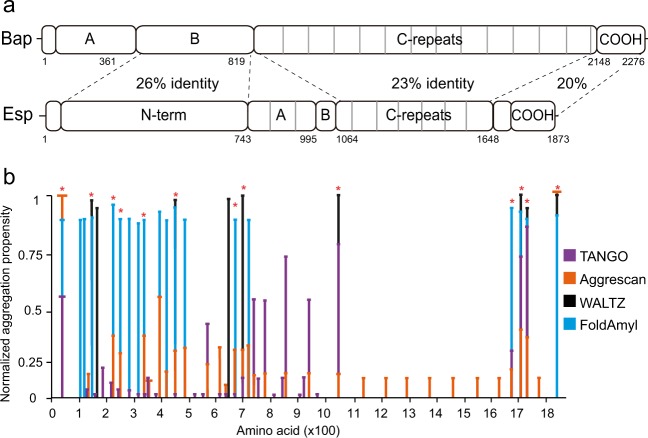
Prediction of the amyloidogenic potential of Esp. **a** Structural similarity between Bap and Esp. The N-terminal domain of Esp (residues 49–743) and region B of Bap (residues 361–819) share 26% sequence identity. The C-repeats of Bap (amino acids 820–2147) and C-repeats of Esp (amino acids 1064–1648) share 23% sequence identity. **b** Comparison of the predicted aggregation propensities. Bars representing the aggregation propensities obtained by four bioinformatic algorithms TANGO (violet), Aggrescan (orange), WALTZ (black), and FoldAmyloid (Blue). Values have been normalized for each predictor. Asterisks denote the amino acid stretches predicted to be amyloidogenic by at least three of the algorithms used.

The enterococcal surface protein Esp possesses high structural similarities to the staphylococcal Bap. The N-terminal domain (Esp_N) of Esp shows 26% identity with the N-terminal domain of Bap (Fig. 1a). Bearing in mind that Bap adopts an amyloid conformation to mediate biofilm formation, we have used four existing computational algorithms commonly used to predict the amyloidogenic propensity of a protein. The selected predictors, including WALTZ^18^, AGGRESCAN^19^, TANGO^20^, and FoldAmyloid^21^, rely on different principles (β-sheet formation, aggregation propensity, hydrogen-bonding interactions, and expected packaging density). The aggregation properties predicted by these algorithms for Esp were normalized and they were compared in Fig. 1b. All the algorithms identified a high number of amyloid-prone segments at the Esp_N region (Fig. 1b and Table 1 and Supplementary Fig. 1A). The alignment of peptides predicted to be amyloidogenic by at least 3 of the 4 algorithms showed no clear conserved domains among them (Supplementary Fig. 1B), suggesting that the presence of aggregation-prone regions rather than amino acid motifs is conserved at the N-terminus of Esp.

**Table 1:** Amino acid sequences corresponding to the predicted amyloid peptides (marked with the asterisks in Fig. 1).

Amino acids	Sequence
21–49	SVGVASVLVGVGLVFGATGIVN
158–174	TNIWKVNYIRANDGLFA
211–221	RLMYRIYLVSH
231–239	IESTGYFLT
323–332	YVSGIQMHMV
440–448	TYGTGYYYLQ
667–686	NDAFSVLDVY
698–705	NTGIVTFT
1050–1058	KGTVVVTYSD
1685–1694	GTMYFWKEKV
1701–1711	NKKATVVVIYP
1718–1726	VEVVISVVD
1843–1865	VDSNIYTIAGGLILLGTLGLLGY

### Esp_N expressed with the curli-dependent amyloid generator (C-DAG) system forms amyloid fibrils

**Fig. 2:**
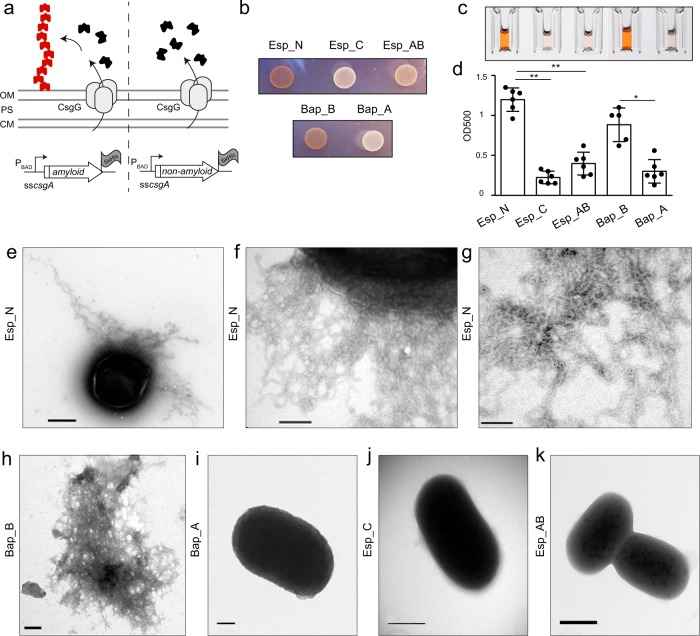
Expression of Esp_N using the curli-dependent amyloid generator (C-DAG) system. **a** Representative scheme of C-DAG. Potential amyloid domains are expressed under the control of the inducible PBAD promoter, which allows the expression of the epitope fused to the first 42 residues of the signal sequence of CsgA. CsgG is expressed under the control of an IPTG-inducible promoter. Outer membrane (OM), cytoplasmic membrane (CM), periplasmic space (PS). **b** Variation in colony color phenotype of *E. coli* cells exporting the N-terminal domain of Esp (Esp_N), a C-repeat (Esp_C), and AB-repeats (Esp_AB) on agar supplemented with CR. *E. coli* expressing Bap_B and Bap_A from *S. aureus* was used as positive and negative controls respectively. **c** CR solubilized using ethanol–acetone from *E. coli* cells expressing Esp_N, Esp_C, Esp_AB, Bap_B, and Bap_A. **d** Quantification of CR. Absorbance was measured at 500 nm. Bars represent the standard deviations of the results of six independent experiments (*n* = 6). Statistically significant differences were determined using Mann–Whitney test *p < 0.05, **p < 0.01. Transmission electron micrographs of negatively stained fiber-like structures formed by *E. coli* cells that express Esp_N **e**, **f** and Bap_B **h**. *E. coli* cells that express Bap_A **i,** Esp_C **j** and Esp_AB **k** did not show the presence of fibers as shown by transmission electron microscopy. Immunogold labeled of fiber-like structures of samples from *E. coli* cells that express Esp_N using anti-His antibodies **g**. Scale bar of panels **e**, **j**, **k** represents 500 nm. Scale bar of panels **f**, **h**, **i** represents 200 nm. Scale bar of panel **g** represents 100 nm.

To explore the amyloid-forming propensity of Esp_N, we used the C-DAG system^22^. Amino acids 67–511 from Esp were cloned into the pExport plasmid that allows the expression of the epitope of interest fused to the first 42 residues of the signal sequence of CsgA (ssCsgA) and the 6xHistidine tag (Fig. 2a). We also cloned into the pExport plasmid a C-repeat of Esp (amino acids 1148–1228), which showed no amyloidogenic-prone region prediction on it, and one A-repeat and part of the B-domain (amino acids 913–1060) that possess only one aggregation-prone peptide (Fig. 1a). Besides, plasmids carrying the *S. aureus* Bap_B or Bap_A regions fused to ssCsgA were included as positive and negative controls, respectively (Fig. 1a). The resulting plasmids were introduced in the carrier *E. coli* VS39 strain, which is deficient in the curli system but expresses *csgG* gene that is required for the protein export. In the presence of arabinose, chimeric proteins are expressed and subsequently guided by the ssCsgA epitope at the extracellular space through a pore-like structure that is formed by the CsgG protein in the outer membrane (Fig. 2a). The presence of extracellular amyloid-like aggregates was detected by analyzing the capacity of the strains to bind Congo Red dye (CR). As shown in Fig. 2b, *E. coli* cells expressing Esp_N domain were able to bind CR as the strain producing Bap_B of *S. aureus*, while no binding to CR was observed in *E. coli* strains expressing Bap_A, Esp_C, and Esp_AB chimeras. We also quantified the CR attached to the cells using a quantitative CR-binding assay. After induction of the proteins using the C-DAG system, *E. coli* cells were incubated in a solution containing CR. The colorant bound to the amyloids-like material was solubilized from the cells using ethanol–acetone (Fig. 2c). Results showed that *E. coli* strain that expressed Esp_N was able to bind significantly higher levels of CR in comparison to *E. coli* strain that express Esp_C and Esp_AB (Fig. 2d). Interestingly, electron microscopy analysis of the *E. coli* strain exporting the Esp_N domain revealed the presence of extracellular dense aggregates surrounding the cells, that are most likely fibrous (Fig. 2e, f), and that specifically reacted with gold-labeled anti-His antibody (Fig. 2g and supplementary Fig. 2). Nonspecific binding was determined in the presence of the secondary gold-labeled antibody (Supplementary Fig. 2). Similar structures were also observed in *E. coli* cells exporting Bap_B (Fig. 2h) but not in cells that expressed Bap_A, Esp_AB, and Esp_C chimeras (Fig. 2i, j, k). Taken together these results showed that the N-terminal domain of Esp expressed in a cell-based aggregation system formed amyloid-like aggregates.

### Recombinant Esp_N assembles into β-sheet-rich structures with amyloidogenic properties at acidic pH

**Fig. 3:**
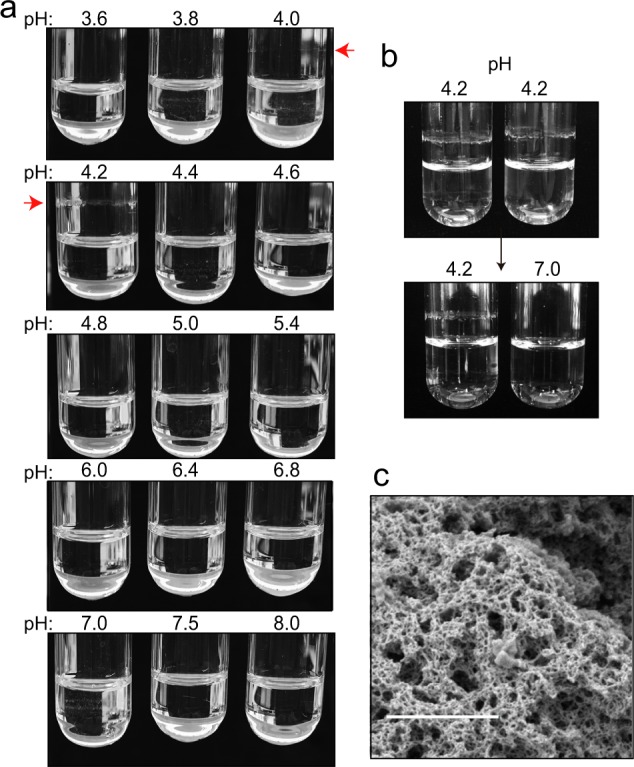
Recombinant rEsp_N protein forms reversible aggregates in acidic phosphate–citrate buffer. **a** 2 μM of purified rEsp_N protein was incubated in phosphate buffer solutions at different pH values. Aggregates started to be visible in buffers with pH 4 and 4.2 (indicated by a red arrow). **b** Reversion assay showed complete disassembly after phosphate–citrate buffer exchange from pH 4.2 to pH 7. **c** Scanning electron microscopy of rEsp_N aggregates. Scale bar represents 1 µm.

To study the biophysical properties of the N-terminal domain of Esp we purified a recombinant Esp_N domain (rEsp_N) (Supplementary Fig. 3). rEsp_N protein was incubated in phosphate–citrate buffer solutions with different pH. As it was previously observed for Bap_B^14^, rEsp_N formed aggregates at acidic pH (pH 4.0–4.2) (Fig. 3a). At pH 4.2, rEsp_N formed a subtle but visible ring of protein adhered to the walls of the tube. Interestingly, the process was reversible and rEsp_N aggregates dissociated completely when the pH raised to 7 (Fig. 3b). Analysis of the structure of the rEsp_N aggregates by scanning electron microscopy showed the formation of a dense mesh-like network (Fig. 3c).

**Fig. 4:**
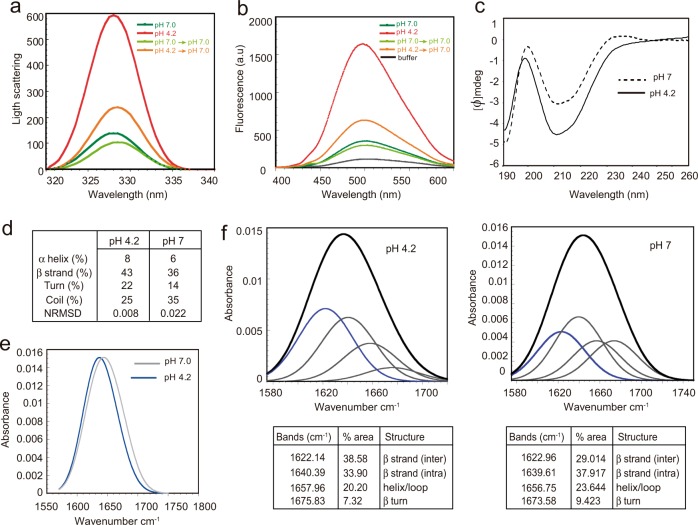
Amyloid features of rEsp_N. **a** Synchronous light scattering of 0.1 mg/ml rEsp_N samples in phosphate–citrate buffer either at pH 7.0, 4.2, and pH-reverted samples to pH 7.0. **b** Bis-ANS fluorescence of 0.1 mg/ml rEsp_N at pH 7.0, pH 4.2, and pH-reverted samples to pH 7.0. Free bis-ANS is represented in black. **c** Far-UV CD spectra of 0.05 mg/ml rEsp_N at pH 7 and pH 4.2. **d** Secondary structure composition of rEsp aggregates. Data were analyzed with Dichroweb implementing the CDSSTR algorithm. **e** FTIR spectra in the amide I region of rEsp_N aggregates formed after 24 h in acidic buffer (blue line) and rEsp_N in its soluble state (black line). **f** Deconvolution of the FTIR spectra of the rEsp_N at pH 4.2 and at pH 7. Component bands after gaussian deconvolution are shown. Percentage of each secondary structure is detailed in the tables.

We determined the relative size of rEsp_N aggregates by static light scattering (Fig. 4a), showing the presence of significantly big aggregates of rEsp_N at pH 4.2 that completely disassembled when buffer was changed to pH 7. We also showed that these self-assembled rEsp_N aggregates bound strongly to bis-ANS dye evidencing their exposure of hydrophobic patches to solvent (Fig. 4b).

In order to test whether the aggregates formed by rEsp_N have amyloid-like properties, we analyzed the secondary structure content by far-UV circular dichroism (CD) of the rEsp_N at pH 4.2 and pH 7. Results showed that rEsp_N domain showed a preponderance of β-sheet content at acidic pH (Fig. 4c, d). We also analyzed the amide I region of the attenuated total reflectance-Fourier transform infrared spectroscopy (ATR-FTIR) spectrum in the range 1700–1600 cm^−1^ of the rEsp_N aggregates (acidic pH) and its soluble state (neutral pH). The overall analysis of both spectra showed a left shift absorbance in the 1610–1630 cm^−1^ range of the β-strand region of the amide I spectrum, which is attributed to amyloid-like β-strand interactions (Fig. 4e). Deconvolution of the spectra showed that the majoritarian band of rEsp_N aggregates was detected at 1620–1630 cm^−1^, which is typically assigned to the presence of inter-molecular β-sheets that contributes 38.5% of the area (Fig. 4f). The dominated structure of the soluble Esp_N (neutral pH) spectra is detected at 1640 cm^−1^ assigned to the presence of intra-molecular β-sheets covering 37.91% of the area. The second majoritarian structures appear at 1640 cm^−1^, covering 33.9% of the area and at 1620–1630 cm^−1^ that contributes 29.01% of the area for the Esp_N aggregates and the soluble protein, respectively. A representation of the second derivative values, which are necessary for the deconvolution method of the bands, is shown in Supplementary Fig. 4. Overall, our data are consistent with the assembly of Esp into supramolecular β-sheet-enriched aggregates.

**Fig. 5:**
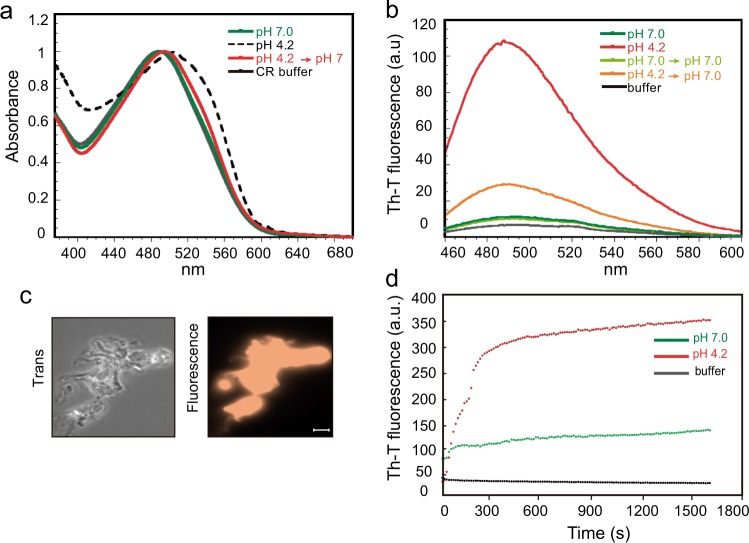
Binding of rEsp_N to amyloid-indicative dyes. **a** Shift in CR absorbance spectrum upon binding of CR dye to 0.1 mg/ml rEsp_N at pH 7.0, pH 4.2, and pH-reverted samples to pH 7.0. Free CR absorbance spectrum is represented in black. **b** Increase in Th-T emission fluorescence upon binding to 0.1 mg/ml of rEsp_N at pH 7.0, pH 4.2, and pH-reverted samples to pH 7.0. **c** Fluorescence of rEsp_N aggregates stained with Proteostat dye. Samples were visualized using a ×100 oil immersion lens. Scale bar represents 10 µm. **d** Aggregation kinetics of rEsp_N at pH 4.2 (red) and at pH 7 (green), as monitored by changes in relative Th-T fluorescence signal. Free Th-T fluorescence is shown in black.

To confirm that the detected β-sheet-enriched aggregates display amyloid-like properties, we analyzed if rEsp_N aggregates could bind amyloid-indicative dyes, such as CR, Thioflavin-T (Th-T), and Proteostat. CR binding to rEsp_N aggregates was determined by analyzing the absorbance recorded from 380 to 680 nm. As it is characteristic for amyloid proteins, incubation of CR in the presence of rEsp_N aggregates formed at pH 4.2 resulted in a red shift of its spectrum compared to the protein incubated at pH 7 and the CR buffer. Moreover, the reversion of the aggregates at pH 7 displayed no red shift in the spectrum (Fig. 5a). Th-T fluorescence emission was enhanced in the presence of rEsp_N aggregates at the intensity of Th-T fluorescence spectral maximum of 488 nm. In contrast, no change in Th-T fluorescence was observed when the protein was incubated at pH 7.0 (Fig. 5b). Finally, staining with Proteostat showed that the dye was significantly concentrated at the rEsp_N aggregates (Fig. 5c). The fact that rEsp_N aggregates bind all these amyloid-indicative dyes confirmed the amyloid-like features of Esp.

In addition, we tested the aggregation kinetics of rEsp_N at pH 4.2 and pH 7 by measuring Th-T fluorescence signal over time. We observed a very high Th-T signal from the very beginning of the reaction when the protein was incubated in acidic buffer. The rapid aggregation of rEsp_N gave a kinetics missing the lag-phase that is typical of the sigmoidal aggregation kinetics of the amyloid proteins (Fig. 5d). Taking together all these results showed that the N-terminal domain of Esp self-assembled in structures with amyloidogenic properties at acidic pH.

### Heterologous expression of Esp_N induces multicellular behavior through the formation of fibrillar structures

**Fig. 6:**
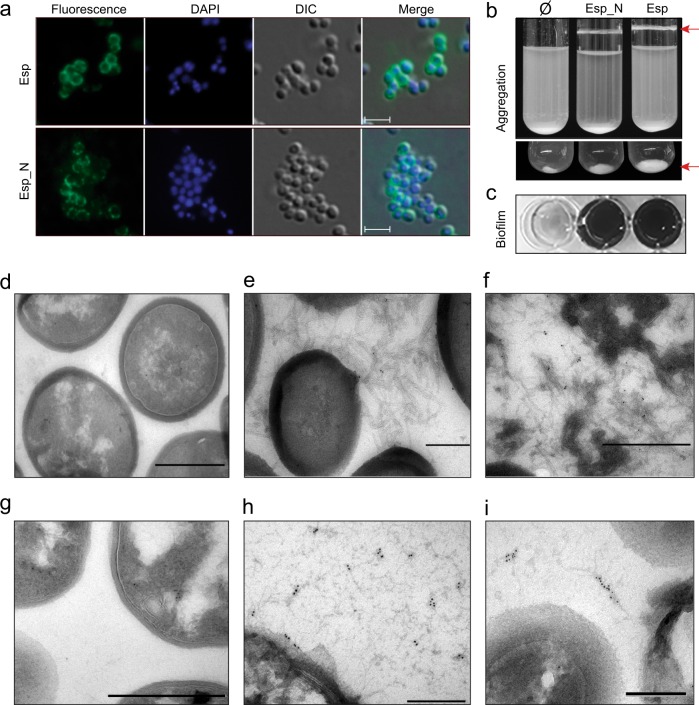
Expression of Esp N-terminal region is sufficient to mediate biofilm phenotype in a heterologous strain. **a** Inmunofluorescence showing surface localization of Esp. Bacteria were fixed and labeled with anti-Esp antibodies and DAPI. Scale bars represent 3 µm. **b** *S. aureus* Δ*bap* expressing chimeric N-terminal domain of Esp (Esp_N) or the entire protein (Esp) forms bacterial clumps (arrows) in overnight cultures grown in TSB-gluc under shaken conditions (200 rpm) at 37 °C. Ø refers to cells complemented with pCN51 empty plasmid. Images of the bottom part of the tubes are shown (bottom panel). **c** Biofilm formation of *S. aureus* Δ*bap* cells that express the N-terminal domain of Esp (Esp_N) or the full protein (Esp). For biofilm formation, bacteria were cultured overnight at 37 °C in microtiter plates under static conditions. Electron micrographs of negatively stained *S. aureus* Δ*bap* **d** and *S. aureus* Δ*bap* expressing Esp_N **e**. **f** Immunogold-labeled samples of fibers formed by amyloid Esp_N. **h, i** Immunogold-labeled electron micrographs of *E. faecalis* 11279 (Esp positive) and its derivative Esp mutant **g**. Scale bar of panels **d**, **f**, **g** represents 500 nm. Scale bar of panels **e**, **h**, **i** represents 200 nm.

We generated a chimeric protein comprising the Esp_N domain (amino acids 50–743) tagged with the 3xFLAG amino acid sequence and linked to the R domain of the clumping factor A (R_ClfA) that contains the LPXTG motif for anchoring to the peptidoglycan. The chimeric protein was expressed from the pCN51 vector under the control of the P_cad-cadC_ promoter. This plasmid was transformed into the biofilm negative *S. aureus* Δ*bap* strain^23^. As a control, we expressed the entire Esp protein from pCN51 plasmid. The localization of both Esp_N-R_ClfA chimeric protein and full Esp on the bacterial cell wall was confirmed by immunofluorescence using anti-esp antibodies (Fig. 6a). As shown in Fig. 6b, bacterial cells expressing the Esp chimeric protein induced cell aggregation (or cell clumping) and biofilm formation similarly to those cells expressing the whole Esp protein. This result confirmed that the N-terminal domain of Esp is sufficient to encourage cell aggregation, similar to the full Esp.

According to the in vitro assays with the rEsp_N protein, Esp-mediated multicellular behavior was only observed under acidic conditions, when bacteria were grown in media supplemented with glucose, TSB-gluc (Fig. 6b, c), or LB-gluc (Supplementary Fig. 5), but not in LB without glucose (pH 7) (Supplementary Fig. 5). Finally, we determined by transmission electron microscopy (TEM) the presence of extracellular material in the cells complemented with the chimeric Esp_N-R_ClfA protein. *S. aureus* Δ*bap* strain expressing the Esp_N-R_ClfA chimera formed a fibrous-like structures that connected cells together (Fig. 6e) and that were not observed in the Δ*bap* cells (Fig. 6d). Moreover, extracellular material positively reacted with gold-labeled anti-Esp antibodies confirming that amyloids fibers are formed by Esp_N-R_ClfA protein (Fig. 6f). Similarly, extracellular fibrous-like structures that reacted with gold-labeled anti-Esp antibodies were detected in the *E. faecalis* 11279 strain that expresses Esp protein (Fig. 6h, i) and not in its derivative Esp mutant (Fig. 6g). Taken together all these results showed that the N-terminal domain of Esp is able to mediate multicellular behavior through the formation of extracellular fibrous-like structures at acidic pH.

### Exogenous addition of rEsp_N to *esp*-negative bacteria induces cell-to-cell aggregation

**Fig. 7:**
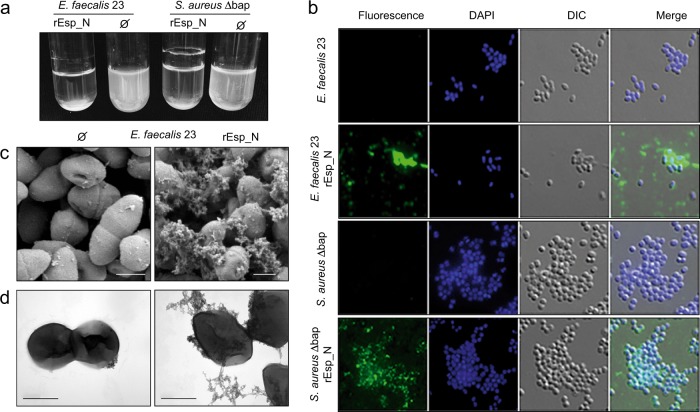
Exogenous complementation of Esp-negative strains with purified rEsp_N. **a** Bacterial clumping of overnight cultures of *esp*-negative *E. faecalis* 23 and *S. aureus* Δ*bap* grown under shaken conditions (200 rpm) at 37 °C in the presence of rEsp_N. Ø indicates no addition of rEsp_N to bacterial culture. **b** Inmunofluorescence showing surface localization of rEsp_N. Bacteria were fixed and labeled with anti-His antibodies and DAPI. Scale bars represent 3 µm. **c** Scanning electron microscopy and **d** transmission electron micrographs of negatively stained *E. faecalis* 23 incubated with (right panel) and without rEsp_N (left panel). Scale bar represents 500 nm.

Purified recombinant amyloid-like proteins usually have biological activity, since they yield multicellular behavior phenotypes undistinguishable from those formed by the wild-type strain when they are exogenously added to biofilm-deficient strains^11,14,24^. To validate the functionality of rEsp_N, we performed an exogenous complementation assay that consisted in determining the capacity of rEsp_N to induce bacterial clumping when exogenously added to a bacterial culture. Specifically, we extracellularly added rEsp_N to a bacterial culture of the *E. faecalis* 23 strain that lack the *esp* gene and therefore is unable to induce bacterial aggregation. As a control, we also used the *S. aureus* Δ*bap* strain. As shown in Fig. 7a, the addition of rEsp_N promoted bacterial aggregation in both strains (Fig. 7a). By using immunofluorescence labeling with anti-His antibodies we were able to detect rEsp_N protein in the clusters formed by both of *E. faecalis* and the *S. aureus* Δ*bap* strains (Fig. 7b). Finally, we analyzed the cell aggregates by SEM and TEM. We detected extracellular tangles of fiber-like material on the cell surface of *E. faecalis* 23 connecting and attaching cells together (Fig. 7c, d). All these results showed that rEsp_N is biologically active and induce cell-to-cell aggregation in *E. faecalis*.

## Discussion

Amyloid fibers have been recently discovered as the main group of proteins composing the extracellular matrix of many unrelated bacteria^5^. Two different types of biofilm-related amyloids machineries are described so far^7^. The first group consists in intrinsic bacterial amyloids, where the amyloid state is the primary functional form of the proteins. Examples of amyloids of this groups are: curli (*csgACB-csgDEFG*) in enterobacteria^25,26^, Fap (*fapA-F*) in *Pseudomonas*^10^ and chaplins in *S. coelicolor*^24^. The second type are facultative bacterial amyloid-like proteins where the protein adopts a functional globular folded structure and depending on proteolytic processing and environmental triggers, it can also switch to an amyloid conformation. It seems that the facultative amyloids play a dual role in biofilm formation acting as adhesins in their native conformation and as matrix scaffolds when they polymerize into amyloid-like fibrillar structures. An example of this type of proteins is Bap of *S. aureus*. Bap is secreted and covalently anchored to the cell wall. At its native conformation, Bap is involved in primary adhesion to an abiotic surface and host cell ligands^27^. Then, Bap is processed releasing to the media fragments containing the N-terminal region to the media, whilst part of the C-terminal repeat region remains anchored to the membrane. When the pH becomes acid the N-terminal domain of Bap switches from its partially ordered native state to an aggregation-prone conformation that facilitates polymerization into amyloid-like fibrillar structures.

The results presented here, propose Esp as a novel facultative amyloid-like protein. The prediction of potentially amyloidogenic regions using several computational algorithms converged to indicate regions capable of adopting an amyloid conformation in the N-terminal domain of Esp (Esp_N). Esp undergoes a limited proteolytic or spontaneous cleavage at certain conditions, as shown by western-blot using antibodies against the N-terminal region of Esp (Supplementary Fig. 6)^14,28^. This processing would enable the release of the N-terminal domain of the protein that self-assembles into amyloid-like fibrillar structures. However, we are unable to determine which part of the N-terminal region of the protein are releasing the processed peptides that form part of the amyloid-like fibers. It is important to notice that sortase-mediated anchoring of Esp is not strictly required for multicellular aggregation, since extracellular addition of rEsp_N-purified protein lacking the LPXTG motif is biologically active and induce cell-to-cell aggregation. Although were able to detect extracellular fibrous dense aggregates in *E. coli* cells exporting the Esp_N domain by electron microscopy analysis, we did not prove in a truthful way that they are canonical amyloid fibers. Secondary structure data (CD spectra and FTIR) and binding to amyloid indicator dyes are not sufficient to affirm that Esp adopts a cross-β arrangement typical of amyloids.

Another coincidence between Esp and Bap is the fact that acidic pH conditions favor amyloid polymerization of both proteins. As occurs with Bap, the pH at which the N-terminal domain of Esp shows aggregation activity (pH 4.2) is close to the isoelectric point of the full protein (p*I* 4.5) and the isoelectric point of the Esp N-terminal domain (p*I* 4.68) and, therefore, lack of net charge at this pH favors the conversion of the protein from its native state to the amyloid-like state. Previous work stablished that high glucose concentration (0.5% (w/v) or higher) in the growth media promoted Esp-mediated biofilm formation without affecting *esp* expression^16,29^. Our results provide a possible explanation to this situation: an acidification of the growth media as a consequence of glucose metabolism would favor Esp amyloid folding and aggregation, thus inducing biofilm formation without relevant changes in *esp* gene expression. Of course, we cannot exclude the possibility that other undetermined factors might participate in this process and interact with Esp. Another major point to be considered in the amyloidogenic capacities of Esp and Bap, is the fact that Esp does not contain any EF-hand motif in its N-terminal domain^30^. For Bap, binding of calcium to the EF-hand motifs is a key process that regulates the formation of amyloid-like fibers. In the presence of calcium, the protein undergoes tertiary rearrangements that increase its stability in solution and thus decrease its aggregation behavior. In case of Esp, this process might be triggered by other metal ions or factors that require further investigation.

Bap and Esp belongs to the biofilm-associated protein (BAP) family. Members of this family have been identified in many unrelated bacteria including BapA of *Salmonella enterica* ser. typhimurium^31^, Bap of *Acinetobacter baumannii*^32^, LapA of *Pseudomonas putida*^33^, BapL of *Listeria monocytogenes*^34^, Bap of *Burkholderia cepacia*^35^, and BpfA of *Shewanella oneidensis*^36^. All the BAP proteins share several structural and functional features (Supplementary Fig. 7). They are high-molecular-weight proteins that contain a core domain of tandem repeats. They play a relevant role in bacterial infectious processes and confer upon bacteria the capacity to form a biofilm^37^. Based on the functional similarities found between Esp and Bap, it is tempting to speculate that other members of the BAP family will also possess amyloid-like properties. In fact, the prediction of potentially amyloidogenic regions using WALTZ algorithm indicated that other BAP members might be capable of forming amyloid-like structures from the N-terminal domain, in the case of BapL of *L. monocytogenes* and from the repeat units, in the case of Bap of *A. baumannii* and BapA from *S. enteritidis* (Supplementary Fig. 7). It is known that repeating units may contain peptides with amyloidogenic behavior^38–40^. In a previous work Lembré et al.^41^, demonstrated that a peptide derived from the repeats of the Bap homolog protein of *S. epidermidis* formed amyloid fibers. This amyloid domain has been proposed to mediate protein–protein interactions and the tertiary structure of Bap in *S. epidermidis*. Although the peptide is repeated 17 times in the protein Bap of the *S. epidermidis* strain C533, the peptide is present only one time in the Bap sequence of *S. aureus*^42^. Our analysis confirmed the absence of amyloid-like domains in the central repeat regions of Bap of *S. aureus* and Esp of *E. faecalis*. Therefore, the presence of several aggregation-prone regions in a domain or the amyloidogenic peptides derived of the repeating units of a protein would be sufficient to promote an amyloid-like conformation of the BAP family of proteins. However, the putative amyloidogenic behavior of all these domains needs to be validated and will be the object of further research that will dig in the mechanistic insight to the likely aggregation mechanism of BAP family proteins.

## Methods

### Oligonucleotides, plasmids, bacterial strains, and culture conditions

Bacterial strains, plasmids, and primers used in this study are listed in Tables 2 and 3. *E. coli* strains were grown in LB broth (Conda-Pronadisa). *E. faecalis* and *S. aureus* strains were grown in trypticase soy broth (Conda-Pronadisa), supplemented with glucose 0.5% (w/v) in *E. faecalis* or glucose 0.25% (w/v) in *S. aureus*, brain heart infusion (BHI) broth and B2 broth media (casein hydrolysate 10 g/l, yeast extract 25 g/l, NaCl 25 g/l, K_2_HPO_4_ 1 g/l, glucose 5 g/l). When required for selection, medium was supplemented with appropriate antibiotics at the following concentrations: erythromycin (Em), 1.5 and 10 µg/ml; ampicillin (Amp), 100 µg/ml; chloramphenicol (Cl), 20 µg/ml.

**Table 2:** Strains and plasmids.

Strains and plasmids		Reference
*E. faecalis* 23	*E. faecalis esp−*, non-biofilm forming	^16^
*E. faecalis* 11279	*E. faecalis esp*+, medium biofilm forming	^16^
*E. coli* VS39	*E. coli* transformed with pVS76 (cat PlacUV5 *csgG*, pACYC184 ori; produces CsgG)	^22^
*E. coli* VS39 Esp_N	VS39 complemented with pEXPORT:*esp_N*	This study
*E. coli* VS39 Esp_C	VS39 complemented with pEXPORT:*esp_C*	This study
*E. coli* VS39 Esp_AB	VS39 complemented with pEXPORT:*esp_AB*	This study
*E. coli* VS39 Bap_B	VS39 complemented with pEXPORT:*bap_B*	^5^
*E. coli* VS39 Bap_A	VS39 complemented with pEXPORT:*bap_A*	^5^
*S. aureus* V329 Δbap	*S. aureus bap*−	^23^
*S. aureus* Δbap Esp_N	*S. aureus* Δbap containing pCN51:*esp_N*	This study
*S. aureus* Δbap Esp	*S. aureus* Δbap containing pCN51:*esp*	This study
*S. carnosus TM400*		^5^
*S. carnosus TM400 Esp_N*	*S. carnosus TM400* containing pCN51:*esp_N*	This study
*S. carnosus TM400 Esp*	*S. carnosus TM400* containing pCN51:*esp*	This study
pET46-eK-LIC_Esp_N	Plasmid for expression of Esp N-terminal	This study
pCN51:*esp_N*	Plasmid for expression of Esp N-terminal domain fused to the R-domain of ClfA, under the control of the Pcad promoter	This study
pCN51:*esp*	Plasmid for expression of Esp under the control of the Pcad promoter	This study
pEXPORT:*esp_N*	Expression vector for C-DAG system including the N-terminal domain of Esp	This study

**Table 3:** Primers.

Primer	Sequence
Esp_Ori_Bam	5′-GGATCCAGGAAGGGAGCAAATTTAGTTTGT-3′
Esp_B_Kpn	5′-GGTACCATTTTTACTTACAGTTACTGCTAAATCG-3′
K-3xF-ClfA	5′-GGTACCGACTACAAAGACCATGACGGTGATTATAAAGATCATGACATCGACTACAAGGATGACGATGACAAGGAAATTGAACCAATTCCAGAGGAT-3’
ClfA-7cE	5′-TTACACCCTATTTTTTCGCC-3′
EF1-InPhu-pCN51-BamH	5′-CGACTCTAGAGGATCCAATAAGGAAGGGAGCAAATTTAGTTTG-3′
Esp2-InPhu-pCN51-KpnI	5′-CTGAATTCGAGCTCGGTACGGAAGAGACTTCTTCCTCTTTTCAGA-3′
EspB-NotI-5	5′-GCGGCCGCACAAGAAAATCCGGGATATACG-3′
EspB-XhoI-3	5′-CTCGAGTTAGTGATGATGGTGATGGTGTGCTAATACAGTACCTTCTTTATCAAC-3′
Esp-Lic-Fw	5′-GACGACGACAAGATGCAAATGGGTGAAG-3′
Esp-Lic-Rv	5′-GAGGAGAAGCCCGGTTAACTGTAAGTAAAAAT-3′

### DNA manipulations and bacterial transformation

General DNA manipulations were performed using standard procedures. Plasmids were purified using the NucleoSpin Plasmid miniprep kit (Macherey-Nagel) according to the manufacturer's protocol. FastDigest restriction enzymes and Rapid DNA ligation kit (Thermo Scientific) were used according to the manufacturer's instructions. Plasmids were transformed into *E. coli XL1-Blue* strain (Stratagene), *E. faecalis*, and *S. aureus* by electroporation. Briefly, *S. aureus* cells were cultured in B2 broth until the OD at 650 nm reaches 0.5. Cells were washed three times with glycerol 10% (v/v). Cells were resuspended in 15 ml of glycerol 10% (v/v) and incubated at 20 °C for 15 min. After incubation, cells were resuspended in 10% glycerol at the final concentration of 1 × 10^10^ CFU/ml. For staphylococcal electroporation, 50 μl of the electrocompetent cells were mixed with 2 μg of plasmid and the mix was added to an ice-cold 0.1-cm gap electrode Gene Pulser Cuvette (Bio-Rad). One pulse was given at 100 Ω, 1.25 V, and 25 F capacity. Immediately after the pulse, 1 ml of SMMP media was added to the cuvette. After incubation at 37 °C for 2 h, the cell mixture was plated onto TSA plates with selective antibiotics. For *E. faecalis*, cells were cultured in BHI media overnight at 37 °C. Cells were washed three times with glycerol 10% (v/v) and were resuspended at the final concentration of 1 × 10^10^ CFU/ml. For enterococcal electroporation, 50 μl of the electrocompetent cells were mixed with 3 μg of plasmid. The mix was added to an ice-cold 0.1-cm gap electrode cuvette. One pulse was given at 400 Ω, 1.25 V, and 25 F capacity. Immediately after the pulse, 1 ml of BHI media with subinhibitory concentration of chloramphenicol (0.2 μg/ml) was added to the cuvette. After incubation at 37 °C for 2 h, the cell mixture was plated onto TSA plates with the selective antibiotic.

### Generation of chimeric proteins

The N-terminal domain of the *esp* gene (Esp_N) was amplified from *E. faecalis* 11279 using primers Esp_Ori_Bam and Esp_B_Kpn (Table 3). To allow anchoring of amplified Esp_N domain to the bacterial cell wall, the R region of clumping factor A (*clfA*) gene containing an LPXTG motif was amplified from *S. aureus* Newman strain using primer K-3xF-ClfA, containing the 3xFlag sequence and a recognition sequence for KpnI, and primer ClfA-7cE with a recognition sequence for EcoRI. The KpnI/EcoRI-restricted R-*clfA* was cloned into pCN51 vector (pCN51:*esp_N-clfA*). The Esp_N-ClfA chimeric protein was expressed under the control of the P_cad-cadC_ promoter, a cadmium inducible promoter. *Esp* gene was amplified using primers EF1-InPhu-pCN51-BamH and EF2-InPhu-pCN51-Kpn and the PCR product was cloned in pCN51 (pCN51:*esp*).

### Construction of plasmids for Esp expression in C-DAG system

To obtain *E. coli* strains for C-DAG system, we PCR amplified the N-terminal region of Esp (*esp_N*) with primers EspB-NotI-5 and EspB-XhoI-3. Nucleotides from 2737 to 3192 for a A-repeat and the B-domain (*esp_AB)*, and nucleotides 3442 to 3685 for a C-repeat of the Esp (*esp_C)* were gene synthetized by GeneArt (Invitrogen, ThermoFisher Scientific). *Esp_N*, *esp_AB*, and *esp_C* were ligated with NotI/XhoI-restricted pEXPORT_XhoI_ plasmid. The final plasmids pEXPORT-Esp_N, -Esp_C and -Esp_AB were transformed in *E. coli* VS39 strain. Induction of protein production and presence of amyloid-like material was assessed on solid medium containing 10 μg/ml CR by evaluating colony color phenotype. To quantify the amount of CR bound to fibers generated from arabinose-induced C-DAG strains, we resuspended and incubated *E. coli* VS39 cells in 1 ml of PBS containing 1.5 μl of CR 0.8% (w/v). The dye was solubilized from the cells using ethanol:acetone (80:20 v/v). Absorbance was measured at 500 nm using a Biospectrophotometer (Eppendorf). Independent experiments were performed six times. Mann–Whitney tests was used to assess significant differences (*p* < 0.05) of absorbance among tested strains.

### Multicellular behavior phenotypes

For the biofilm formation assay strains were grown overnight at 37 °C and then diluted 1:40 in the corresponding media. Cell suspension was used to inoculate sterile 96-well polystyrene microtiter plates (Thermo Scientific). After 24 h of incubation at 37 °C wells were gently rinsed two times with water, dried, and stained with crystal violet for a few minutes. To quantify biofilm formation capacity, crystal violet adhered at the bottom of the wells was resuspended with 200 μl of a solution of ethanol:acetone (80:20 v/v) and the absorbance was quantified at 595 nm.

### Protein expression and production of anti-Esp antibodies

N-terminus of Esp (amino acids 50–743) was PCR amplified from *E. faecalis* 11279 using high fidelity Phusion DNA Polymerase (Thermoscientific) and primers Esp-LIC-Fw and Esp-LIC-Rv (Table 3) designed for use in the LIC cloning system. The resulting fragment was cloned in pET46-Ek/LIC vector (Novagen). Overnight cultures of *E. coli* BL21 DE3 containing the expression plasmid were diluted 1:100 and grown to an OD_600nm_ of 0.6. Isopropyl B-d-thiogalactopyranoside (IPTG) was added to a final concentration of 0.4 mM and the cultures were shaken (150 rpm) at 18 °C overnight. After centrifugation, pellets were resuspended, sonicated, and centrifuged. Supernatants were filtered (0.45 μm) and rEsp_N protein purified by Ni affinity chromatography using HisTrap^TM^ FF columns (GE Healthcare). To achieve the highest purity, size exclusion chromatography was applied with a HiLoad 16/600 Superdex 200 pg column (GE Healthcare). The concentration of the purified protein was determined by the bicinchoninic acid (BCA) Protein Assay (Pierce, Thermo Scientific) using BSA as standard. Chicken polyclonal antibodies raised against purified rEsp_N protein were supplied by Davids Biotechnologie company. Antibodies were subsequently purified by chromatography.

### Immunoblot analysis

One milliliter of an overnight culture was harvested, washed, and finally resuspended in 100 μl of PBS buffer containing 30% (w/v) raffinose (Sigma), 5 μl of lysostaphin 1 mg/ml (Sigma), and 2 μl of DNase 1 mg/ml (Sigma). After 2 h of incubation at 37 °C, cells were centrifuged. The supernatants from surface protein extracts were recovered and analyzed by SDS–PAGE (7.5% (w/v) separation gel; 5% (w/v) stacking gel). For Western blot analysis, protein extracts were blotted onto Hybond-ECL nitrocellulose membranes (Amersham Biosciences). Anti-Esp-purified antibody was diluted 1:10,000 with PBS-Tween 5% (w/v) skim-milk. Horseradish peroxidase-conjugated goat anti-chicken (Abcam Ab97135) diluted 1:20,000 in PBS-Tween 5% (w/v) skim-milk was used as a secondary antibody for Esp detection and the subsequent chemiluminescence reaction was recorded.

### Formation of rEsp_N aggregates and reversion assay

To determine the effect of the pH in protein aggregation, rEsp_N recombinant protein at a final concentration of 2 μM was incubated in phosphate-citrate buffer at pH ranging from 3.6 to 8. Macroscopy protein aggregates were visualized as a ring of protein adhered to the glass wall at the air–liquid interface after 24 h of incubation at 37 °C under shaking conditions (200 rpm). For reversion assay of rEsp_N aggregates formed after 24 h of incubation at 37 °C, the phosphate–citrate buffer at pH 4.2 was removed and exchanged for phosphate–citrate buffer at pH 7. After an overnight incubation at 37 °C and shaking conditions, dissolution of rEsp_N aggregates was macroscopically determined.

### Th-T binding

rEsp_N protein at 0.1 mg/ml was incubated at pH 7.0, pH 4.2, or pH-reverted from pH 4.2 to pH 7.0 and Th-T added at 25 µM final concentration before measuring. Fluorescence emission spectra were recorded in the range of 460–600 nm with an excitation wavelength of 445 nm using a 5 nm slit width for excitation and emission in a Jasco FP-8200 spectrophotometer (Jasco Corporation, Japan) at 25 °C. Aggregation kinetics of 0.05 mg/ml rEsp_N protein in phosphate–citrate buffer at pH 4.2 and pH 7.0 were recorded for 1800 s under agitation (800 rpm) at 25 °C, in the presence of 25 μM Th-T. The kinetic traces were measured exciting at 440 nm and emission was recorded at 475 nm; 5 nm slit widths were used for excitation and emission in a Jasco FP-8200 spectrophotometer.

### CR and ProteoStat binding

Binding of CR to 0.2 mg/ml rEsp_N protein was evaluated at pH 7.0, pH 4.2, and with pH-reverted samples from pH 4.2 to pH 7.0. rEsp_N samples were diluted to a final concentration of 0.1 mg/ml in buffer at pH 7.0 and incubated in the presence of 10 µM CR dye. After 5 min of equilibration, the absorbance spectra were recorded from 375 to 700 nm in a Specord200 Plus spectrophotometer (Analytik Jena, Germany).

For ProteoStat staining, 2 μM aggregated rEsp_N at pH 4.5 were incubated for 30 min, at room temperature and in darkness with ProteoStat Mix buffer (1X Assay Buffer, 1 μl ProteoStat). Preparations were observed with an Axioskop 2 plus epifluorescence microscope (Zeiss) using an excitation setting of 550 nm and an emission setting of 600 nm. Images were acquired and analyzed with EZ-C1 software (Nikon).

### Far-UV CD

Far-UV CD spectra were measured on a Chirascan (Applied Photophysics) spectropolarimeter thermostated at 25 °C. rEsp_N (10 mg/ml) in 20 mM Tris–HCl pH 8, 50 mM NaCl was diluted 200-fold to 0.05 mg/ml in 10 mM MOPS at either pH 4.2 or 7.0. Spectra were recorded from 260 to 190 nm, at 0.2 nm intervals, 1 nm bandwidth, and a scan speed of 50 nm/min. Ten accumulations were averaged for each spectrum. Deconvolution of data was performed using the Dichroweb server implementing the CDSSTR algorithm.

### Static light scattering

Fluorescence spectra were analyzed for rEsp_N samples diluted at 0.1 mg/ml in phosphate–citrate buffer either at pH 7.0 or 4.2, and with samples reverted from pH 4.2 to pH 7.0. Scattering was measured exciting at 330 nm and recording in the 320–340 nm range, using excitation and emission bandwidths of 5 nm at low sensitivity, in a Jasco FP-8200 fluorescence spectrophotometer (Jasco Corporation, Japan).

### Bis-ANS binding

Bis-ANS-binding assay was performed incubating 0.1 mg/ml rEsp_N (stock 0.2 mg/ml) in phosphate–citrate buffer at pH 4.2 and 7.0 containing 10 µM bis-ANS. Samples were measured in a Jasco FP-8200 spectrophotometer (Jasco Corporation, Japan) at 25 °C, in the 400–600 nm range with an excitation wavelength of 370 nm using a slit width of 5 nm for excitation and emission.

### ATR-FTIR spectroscopy

ATR FTIR spectroscopy analyses of Esp_N samples in phosphate–citrate buffer pH 4.2 and in buffer pH 7 were performed with a Bruker Tensor 27 FTIR Spectrometer (Bruker Optics Inc.) supplied with a Golden Gate MKII ATR accessory. Each spectrum consists of 32 acquisitions measured at a resolution of 1 cm^−1^. Spectra were acquired, background subtracted, baseline corrected, and normalized, with the Min/Max normalization method, using the OPUS MIR Tensor 27 software. For the analysis of the IR spectra, the second derivative was calculated and plotted to define the number and position of band components under the absorbance spectra, according to the local minima. Next, spectra were smoothed using the non-parametric smoother Loess at a 5% level and automatically fitted with the “Peaks I residuals” method using PeakFit package v4.12 (Systat Software), placing initially as many peaks as local minima appeared in the secondary derivative of the absorbance spectra. The resulting Gaussian curves were plotted using the amplitude and center values of the fitted bands. In order to assign secondary structures to band components, frequencies in the range 1611–1630 cm^−1^ were assigned to intermolecular β-sheet structures, 1630–1640 cm^−1^ to intramolecular β-sheet structures, 1648–1657 cm^−1^ to β-helical structures and 1662–1687 cm^−1^ to β-turns, as described in the literature.

### Immunofluorescence

Cells were grown overnight in the corresponding tested conditions and fixed during 10 min with 3% (v/v) paraformaldehyde (SIGMA). 200 μl of fixed bacteria were placed on coverslips and incubated for 30 min. After several washes with phosphate-buffered saline (PBS), cells were saturated with PBS–0.5% (w/v) BSA, and finally stained with anti-Esp or monoclonal anti-polyHistidine antibody (Sigma Aldrich H1029) antibodies diluted 1:1000. Alexa Fluor 488-conjugated goat anti-chicken (Abcam ab150169) or Alexa Fluor 488-conjugated goat anti-mouse (Invitrogen A-11029) diluted 1:200 were used as a secondary antibody and DAPI diluted 1:200 was used to label bacteria.

### Microscopy analysis

For TEM, cells were grown overnight in the corresponding tested conditions, washed twice with PBS and then fixed with 2% (v/v) paraformaldehyde (SIGMA) for 1 h at room temperature. Formvar/carbon-coated nickel grids were deposited on a drop of fixed sample during 5 min and rinsed three times with PBS. Negative staining was performed using 2% (v/v) uranyl acetate (Agar Scientific, Stansted, UK). Observations were made with a JEOL 1011 transmission electron microscope. For Esp immunogold labeling, grids coated with the sample were washed and incubated for 45 min on a drop of PBS containing 1:10 antibody against Esp. After washing with PBS, grids were incubated for 45 min with gold-conjugated (6 nm) goat-anti-chicken secondary antibody (Abcam Ab3960). For 6xHis immunogold labeling, samples were incubated with monoclonal anti-polyHistidine antibody (Sigma Aldrich H1029) (1:10). After washing with PBS, grids were incubated with gold-conjugated (10 nm) goat anti-mouse IgG (H&L) secondary (AURION 810.022). Grids were stained with 2% (v/v) uranyl acetate as described above. For scanning electron microscopy, cells on thermanox coverslips were fixed for 1 h in 0.07 M sodium cacodylate buffer (pH 7.3) containing 1.2% glutaraldehyde and 0.05% Ruthenium red (RR). The samples were then washed in the same buffer containing 0.05% RR and post‐fixed in 1% osmium tetroxide in cacodylate buffer. Coverslips were treated with the critical point drying method and observed on a Zeiss JSM 982 scanning electron microscope at the Laboratoire de Biologie Cellulaire et Microscopie Electronique, UFR Medecine (Tours, France).

### Reporting summary

Further information on experimental design is available in the Nature Research Reporting Summary linked to this paper.

## Supplementary information

Supplementary Information

Reporting Summary

## Data Availability

The data that support the findings of this study are included in the article, its supplementary information files, or are available from the corresponding author upon reasonable request.
