# Supplementary material for: The biofilm-associated surface protein Esp of Enterococcus faecalis forms amyloid-like fibers
Source: NPJ Biofilms Microbiomes. 2020 Mar 27;6:15. doi: 10.1038/s41522-020-0125-2 (PMC7101364; doi:10.1038/s41522-020-0125-2)
Supplement: Supplementary file 1 — Supplementary Information [file 41522_2020_125_MOESM1_ESM.pdf]

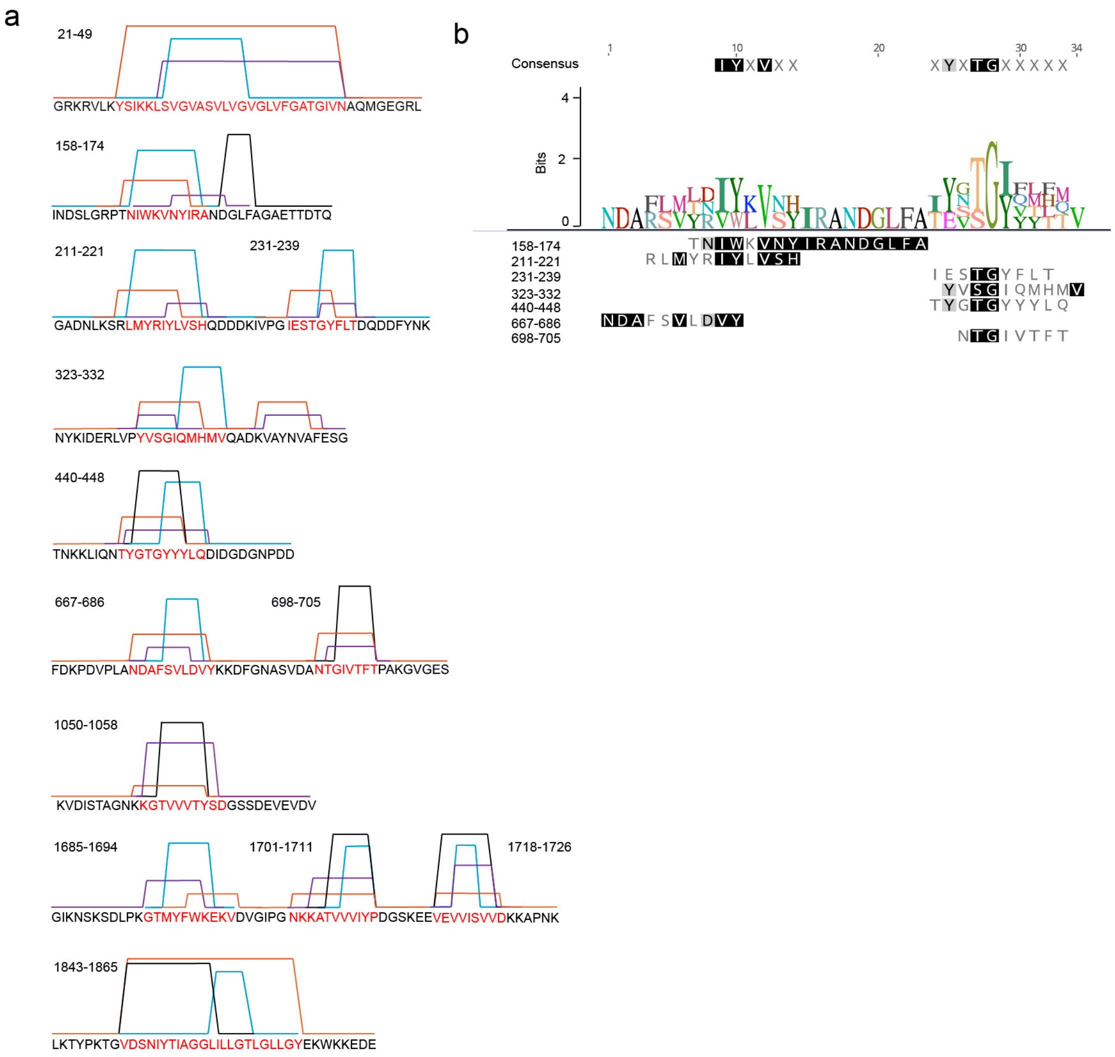

Supplementary Fig 1. a) Mapping of amyloidogenic regions prediction that match with at least 3 algorithms. TANGO (violet), Aggrescan (orange), WALTZ (black) and FoldAmyloid (Blue). b) Alignment of the amino acids with aggregation propensity of the N-terminal domain of Esp using Geneious Prime.

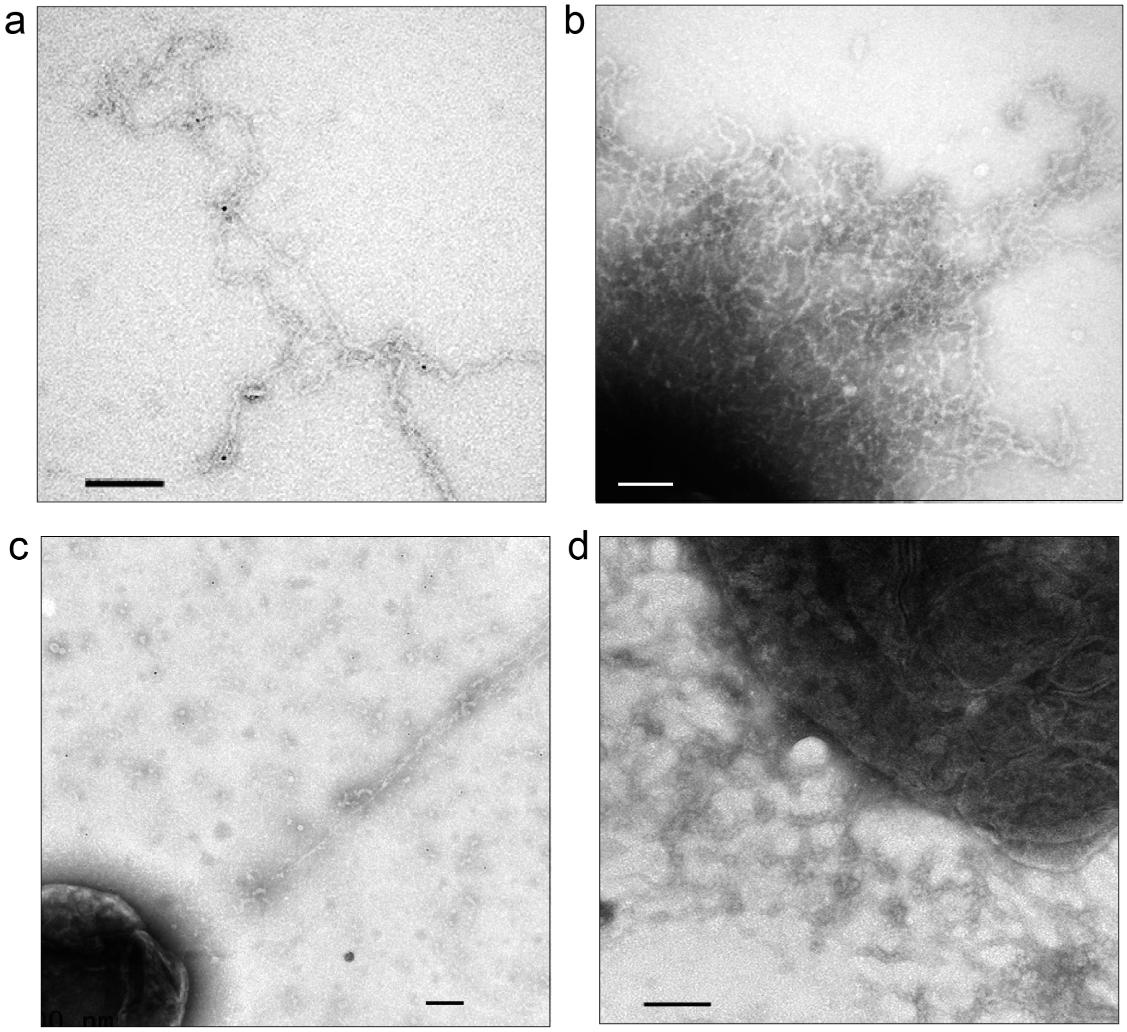

Supplementary Fig. 2. a-b) Immunogold labeled of fiber-like structures of samples from *E. coli* cells that express Esp_N using anti-His antibodies. c-d) Control of non-specific binding was determined in the presence of the secondary gold-labelled antibody. Scale bar of panels a, b represents 100 nm. Scale bar of panels c, d represents 200 nm.

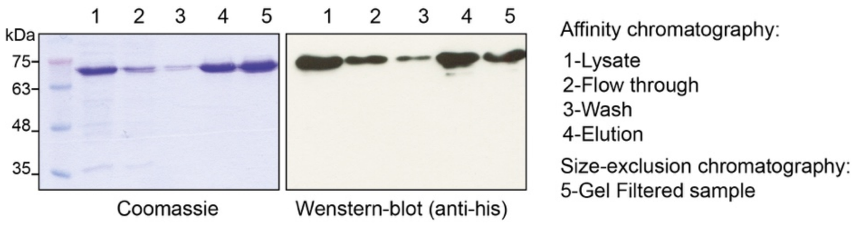

Supplementary Fig. 3. SDS-PAGE and Western blot analyses of rEsp_N protein purified from *E. coli* BL21 strain. The different fractions obtained during the purification process are indicated with numbers. Proteins were separated using 12% acrylamide gels, stained with Coomassie brilliant blue stain and probed with anti-histidine antibodies. All blots were derived from the same experiment and were processed in parallel.

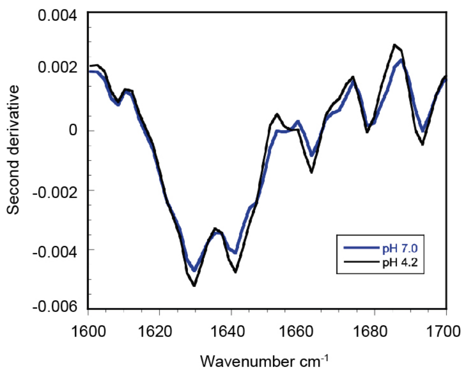

Supplementary Fig. 4. The secondary-derivative spectrum of rEsp_N at pH 4.2 (black) and at pH 7 (blue).

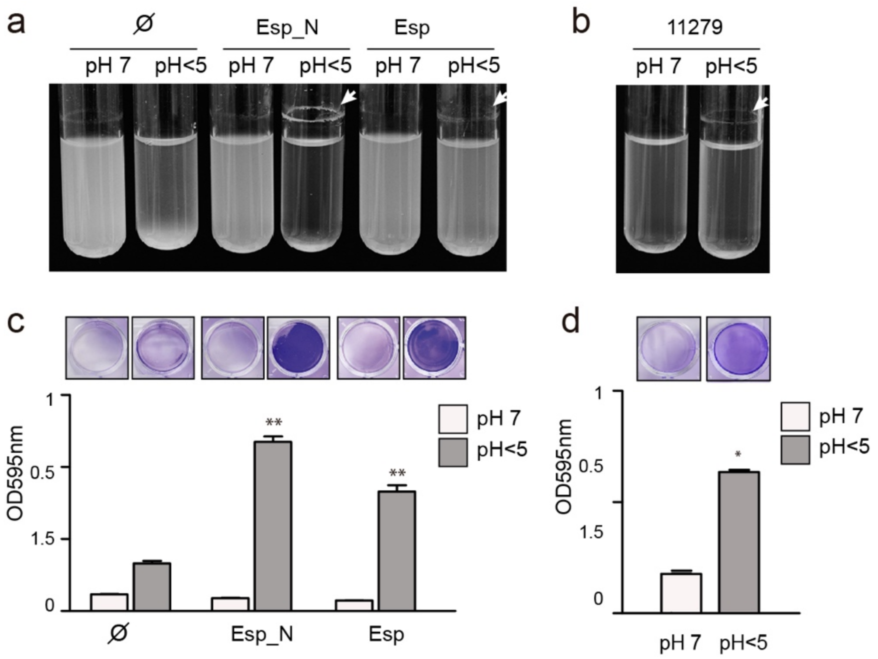

Supplementary Fig. 5. The effect of pH on Esp-mediated aggregation and biofilm formation. a) *S. aureus* Δbap expressing chimeric N-terminal domain of Esp (Esp_N) or the entire protein (Esp) and *E. faecalis* strain 11279 that expresses Esp (b), formed bacterial clumps (arrows) in overnight cultures grown in LB (pH 7) or LB supplemented with glucose (pH <5) under shaken conditions (200 rpm) at 37°C. Ø refers to cells complemented with pCN51 empty plasmid. c) Biofilm formed by *S. aureus* Δbap cells that express the N-terminal domain of Esp (Esp_N) or the full protein (Esp) and biofilm formed by the *E. faecalis* strain 11279 (d). For biofilm formation, bacteria were culture grown in LB (pH 7) or LB supplemented with glucose (pH <5) overnight at 37 ºC in microtiter plates under static conditions. The bacterial cells were stained with crystal violet, and biofilms were quantified by solubilizing the crystal violet with alcohol-acetone and determining the absorbance at 595 nm. The error bars represent the standard deviation of the results of three repetitions. ** $P < 0.01$; *, $P < 0.05$.

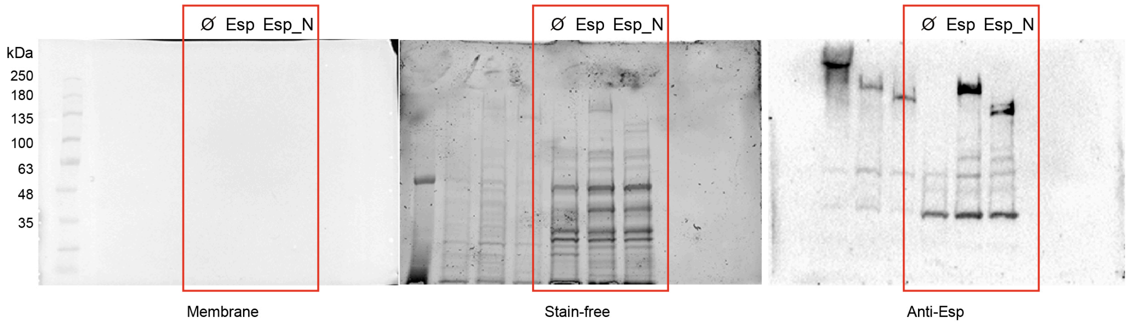

Supplementary Fig. 6. Western immunoblotting results showing cell surface protein patterns of *S. carnosus* TM400 cells that express the full Esp protein (lane 2) or N-terminal domain of Esp fused to ClfA-R domain (lane 3). Cell surface proteins from bacteria with empty plasmid are load in lane 1. Cell wall proteins extracted from exponential cultures were separated on 7.5% (w/v) acrylamide gels and probed with anti-Esp antibodies. Cell wall proteins extracted from exponential cultures were separated on 7.5% (w/v) acrylamide gels and probed with anti-Esp antibodies. All blots were derived from the same experiment and were processed in parallel.

a

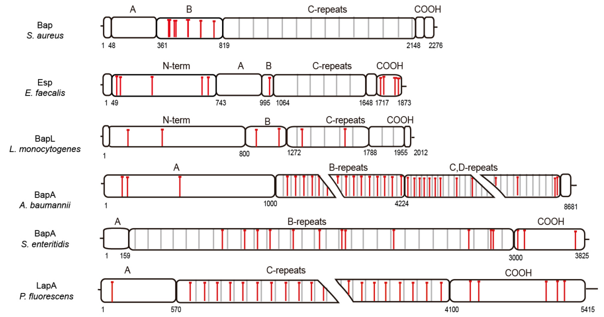

b

	B_Bap_ S.aureus	N_Esp_ E.faecalis	N_BapL_ L.monocyt	A_BapA_ S.enteritidis	A_Bap_ A.bauman	A_LapA_ P.fluoresc
B_Bap_S.aureus	100%					
N_Esp_E.faecalis	26.2%	100%				
N_BapL_L.monocytog	7.5%	6.6%	100%			
A_BapA_S.enteritidis	7.2%	10.7%	4.2%	100%		
A_Bap_A.baumannii	7.9%	11.1%	5.6%	8.4%	100%	
A_LapA_P.fluorescens	3.7%	4.8%	7.5%	6.4%	10.7%	100%

c

	B_Bap_ A.bauman	C_Bap_ S.aureus	B_BapA_ S.enteritidis	A_LapA_ P.fluoresc	C_Bap_ A.bauman	C_BapL_ L.monocyt	C_Esp_ E.faecalis
B_Bap_A.baumannii	100%						
C_Bap_S.aureus	39.8%	100%					
B_BapA_S.enteritidis	26.7%	30.6%	100%				
A_LapA_P.fluorescens	14.3%	16.1%	10.8%	100%			
C_Bap_A.baumannii	14.3%	8.5%	9.1%	7.8%	100%		
C_BapL_L.monocytog	8.6%	7.7%	8.4%	8.2%	10.4%	100%	
C_Esp_E.faecalis	11.0%	10.7%	16.1%	14.3%	13.2%	6.9%	100%

d

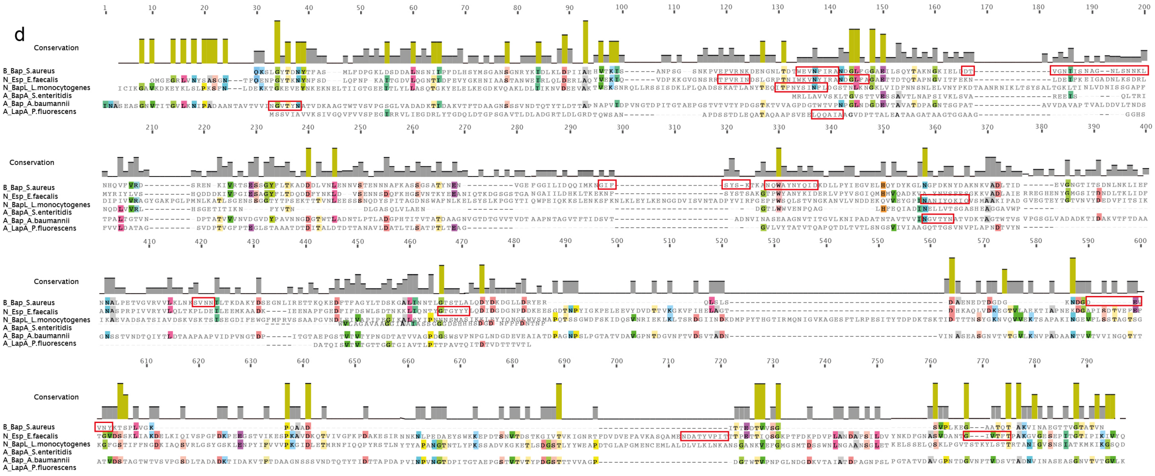

e

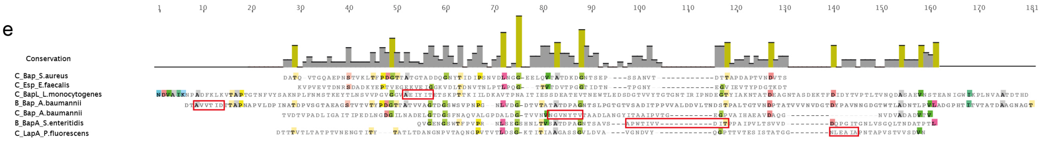

Supplementary Fig. 7 a) Schematic representation of different proteins belonging to the Bap family. The diagrams show the different domains and length of Bap from *S. aureus*, Esp from *E. faecalis*, BapL from *L. monocytogenes*, BapA from *A. baumanii*, BapA from *S. enteritidis* and LapA from *P. fluorescens*. Red vertical lines within each diagram indicate predicted peptides with potential amyloid-like properties. Prediction was performed using Waltz algorithm. b) Pairwise percentage identity matrix of the N-terminal domains of Bap proteins from different bacterial species. c) Pairwise percentage identity matrix of a C-repeats of each Bap proteins. The matrices were obtained from corresponding multiple sequence alignments done using *Geneious Prime* algorithm. d) Protein sequence alignment of N-terminal regions of Bap proteins from the different bacterial species. e) Protein sequence alignment of one repeat of each Bap protein. The alignments were generated using *Geneious Prime* multiple sequence alignment tool. Amyloid-prone regions predicted by Waltz algorithm are highlighted by red rectangles.
